# Gold nanoparticles-induced cytotoxicity in triple negative breast cancer involves different epigenetic alterations depending upon the surface charge

**DOI:** 10.1038/s41598-018-30541-3

**Published:** 2018-08-16

**Authors:** Sunil Kumar Surapaneni, Shafiya Bashir, Kulbhushan Tikoo

**Affiliations:** Laboratory of Epigenetics and Diseases, Department of Pharmacology and Toxicology, National Institute of Pharmaceutical Education and Research (NIPER) S.A.S, Nagar, India

## Abstract

Gold nanoparticles (AuNPs) are used enormously in different cancers but very little is known regarding their molecular mechanism and surface charge role in the process of cell death. Here, we elucidate the molecular mechanism by which differentially charged AuNPs induce cytotoxicity in triple negative breast cancer (TNBC) cells. Cytotoxicity assay revealed that both negatively charged (citrate-capped) and positively charged (cysteamine-capped) AuNPs induced cell-death in a dose-dependent manner. We provide first evidence that AuNPs-induced oxidative stress alters Wnt signalling pathway in MDA-MB-231 and MDA-MB-468 cells. Although both differentially charged AuNPs induced cell death, the rate and mechanism involved in the process of cell death were different. Negatively charged AuNPs increased the expression of MKP-1, dephosphorylated and deacetylated histone H3 at Ser10 and K9/K14 residues respectively whereas, positively charged AuNPs decreased the expression of MKP-1, phosphorylated and acetylated histone H3 at Ser 10 and K9/K14 residues respectively. High-resolution transmission electron microscopy (HRTEM) studies revealed that AuNPs were localised in cytoplasm and mitochondria of MDA-MB-231 cells. Interestingly, AuNPs treatment makes MDA-MB-231 cells sensitive to 5-fluorouracil (5-FU) by decreasing the expression of thymidylate synthetase enzyme. This study highlights the role of surface charge (independent of size) in the mechanisms of toxicity and cell death.

## Introduction

Breast cancer is the most common invasive cancer worldwide and for triple negative breast cancer, no targeted effective therapy is available till date^1,2^. Resistance to anticancer drugs and their side effects are the major hurdles in the treatment of TNBC. Nanotechnology, a rapid progressing field in the area of cancer therapy and diagnosis has the potential to impact medicine, refine quality of life, and eventually improve patient outcomes^3^. Particularly, the small size of nanoparticles (1–100 nm) facilitates their easy access to a wide range of cells and tissues and also their surface can be modified with desired ligands and receptors to specifically target cells of interest as well as to achieve controlled drug release^4^. Biocompatible, non-toxic and efficient nanoparticle carriers also intrigues new hopes for the gene therapy of cancer^5^. Among these, gold nanoparticles (AuNPs) are being used successfully till now as versatile, selective and highly multifunctional anti-cancer therapeutics due to their unique physicochemical properties, therapeutic payload efficiency of drugs, biological compatibility, theranostic applications and radiation sensitizer effects^6–8^. Their surface can be functionalised with various biomolecules, thereby inciting specific functions, selectivity to the targets and stability in biological environments. AuNPs are also used as pharmaceutical cargos for targeted delivery of anticancer drugs like methotrexate and chloroquine^9,10^. AuNPs caused caspase-3 activation, DNA fragmentation and also increased the expression of cleaved PARP and cleaved caspase 9 in MDA-MB-231 cells^11,12^. Many reports have shown that oxidative stress is responsible for AuNPs induced cytotoxicity in various tumors. Oxidative stress in turn causes mitochondrial dysfunction, MAPkinases activation and DNA damage and eventually apoptosis of cancerous cells^13–15^. Surface chemistry (i.e., related to size, charge and zeta potential) of nanoparticles plays a crucial role in mediating the biological effects within the living cells^16–18^. An *in vivo* long term study conducted on mice with gold nanoclusters for 90 days (5.9 mg/kg, i.p.) revealed that negatively charged ones are highly effective, as they displayed lower renal excretion and increased uptake by the tumour cells^19^. Recently, an *in vitro* long term study on human dermal fibroblast cells provides evidence that cells can adaptively respond to chronic, low-level AuNPs treatment, as well as the cell stress response is sustained even if the gold nanoparticles are removed after acute exposure^20^. However, very little is known regarding the sustained stress response even after removal of AuNPs and also the molecular mechanism involved in the process of cell death. Paucity of the data regarding this made us to elucidate and unravel how AuNPs induce cell death to triple negative cancer cells. In addition to this, we also tried to understand the role of surface charge of AuNPs in the process of cell death of triple negative breast cancer cells. Understanding of this will be of profound clinical significance for AuNPs either as stand-alone therapeutics or in combination with anti-cancer drugs.

## Methods

### Cell culture

MCF-10A, MDA-MB-231 and MDA-MB-468 cells were obtained from American Type Cell Culture (ATCC, Manassas). Cells were grown under standard conditions of 5% CO_2_ and 37 °C in a controlled humidified incubator. MDA-MB-231 and MDA-MB-468 cells were cultured in Dulbecco’s Modified Eagle’s Medium (DMEM) (Sigma Aldrich) supplemented with 10% FBS and 1% penicillin-streptomycin (GIBCO, USA). MCF-10A cells were cultured in DMEM media supplemented with Horse serum, EGF (100 mg/mL), Hydrocortisone (1 mg/mL), Insulin (10 mg/mL), Cholera toxin (1 mg/mL) and 1% penicillin-streptomycin. All the cells were used prior to passage 20. Cells were routinely passaged using 0.25% trypsin/0.1% EDTA. All the other chemicals were purchased from Sigma (St. Louis, MO, USA), unless otherwise mentioned.

### Cytotoxicity assay

Cytotoxicity (MTT) assay for AuNPs was carried out in MDA-MB-231, MDA-MB-468 and MCF-10A cells according to the method as described^21^. Cells were harvested, counted and seeded at a density of 5000 cells per well in a 96 well plate and then incubated for 24 h. Then cells were treated with synthesized AuNPs of various concentrations ranging from 25 µg/mL to 1000 µg/mL for 24 h. 2.5 mg of MTT (3-[4, 5-dimethylthiazol-2-yl]-2,5-diphenyltetrazolium bromide) was dissolved in 500 µL of phosphate-buffered saline (PBS) and diluted to 5 mL with serum free DMEM medium. After 24 h of AuNPs treatment, 200 μL of the MTT solution was added to individual wells. The plate was then wrapped in aluminium foil and incubated at 37 °C for 4 h. The solution in each well, containing media, unbound MTT and dead cells, was removed by suction. 200 μL of DMSO was added to each well. The plate was then kept for shaking and then absorbance was measured at a dual wavelength of 550 nm and 630 nm using a multi-mode automated micro plate reader (Flex station III, Molecular Devices, Sunnyvale, CA, USA). The results were expressed as percentage cell viability, assuming the viability of control cells as 100%. Three independent experiments were performed for each study and all measurements were performed in triplicate.

### Intracellular detection of oxidative stress

We investigated intracellular ROS generation in MDA-MB-231 and MCF-10A cells according to the method as described^22^. CM-H2DCFDA used for this assay was purchased from Thermo Fisher Scientific. Cells seeded in 6-well plates (3 × 10^5^ cells per well) were allowed to grow like as mentioned above for 24 h. Cells were then treated with 100 µg/mL, 250 µg/mL and 500 µg/mL of AuNPs for 30 min. Afterwards, media was removed from the wells and PBS washing was carried out. Then, 1 ml CM-DCFDA solution (1 µL CM-DCFDA/ ml PBS) was added to the wells. After 10 min of incubation, DCFDA solution was removed and the cells were washed with PBS and finally observed under microscope for florescence.

### Chemosensitivity assay

MDA-MB-231 cells seeded in 24 well plates (1 × 10^5^ cells per well) were allowed to grow like as mentioned above. Cells were then treated with synthesized AuNPs of 500 µg/mL for 12 h. Afterwards, media was removed and PBS washing was carried out in AuNPs treated groups and were then treated with 5-Fluorouracil (25 µM) for another 12 h and finally cell proliferation was assessed. Thymidylate synthetase mRNA expression was also checked out in these treated cells.

### Statistical analysis

All the values were expressed as mean ± S.E.M. Statistical comparison among more than two different groups was performed using one-way analysis of variance (ANOVA) followed by Tukey’s test. P value less than 0.05 was considered to be significant.

## Results

### Gold nanoparticles (AuNPs) exert cytotoxic effects in MDA-MB-231 cells through the induction of oxidative stress

**Figure 1:**
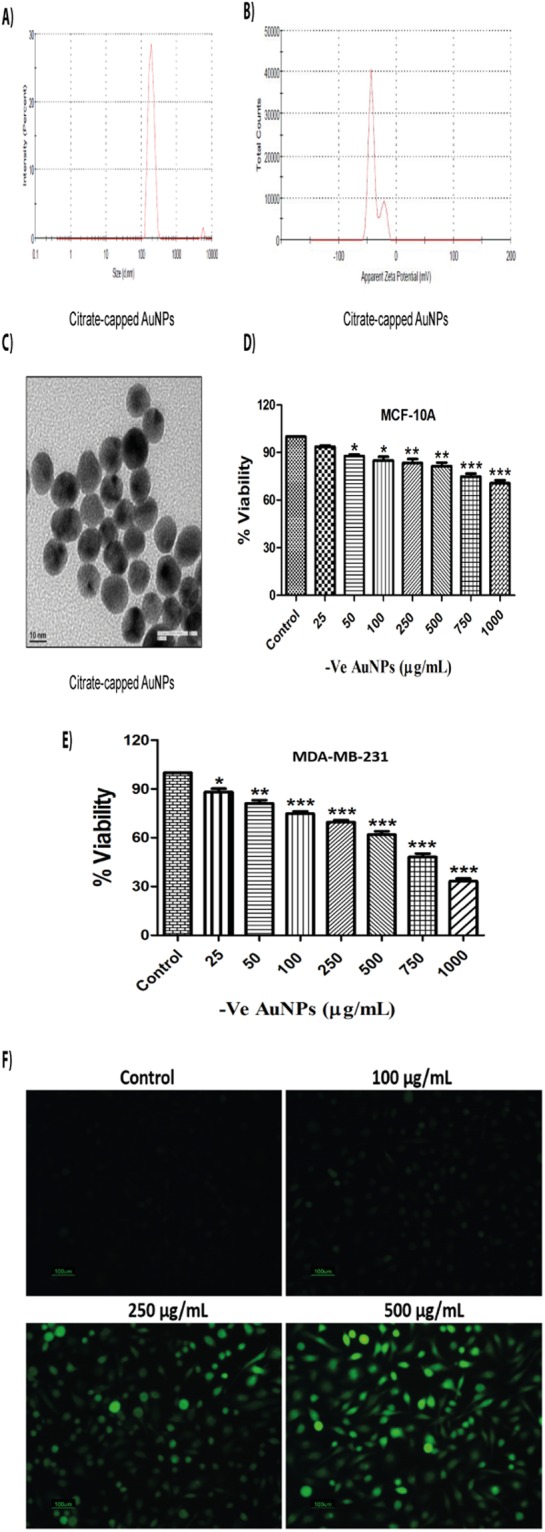
Citrate-capped AuNPs exert cytotoxic effects in MDA-MB-231 cells through the induction of oxidative stress. (**A**) Zeta (Average size) of citrate-capped AuNPs (**B**) Zeta potential of citrate-capped AuNPs (**C**) TEM image of citrate-capped AuNPs (**D**) MTT assay of citrate-capped AuNPs in MCF-10A cells for 24 h (**E**) MTT assay of citrate-capped AuNPs in MDA-MB-231 cells for 24 h (**F**) DCFDA assay of citrate-capped AuNPs at concentrations of 100, 250 and 500 μg/mL in MDA-MB-231 cells respectively for 30 min. Results shown are representative of three different experiments. All values were expressed as mean ± S.E.M. *p < 0.05, **p < 0.01, ***p < 0.001 significant vs control.

Synthesized AuNPs were characterised by several means such as zeta size, zeta potential analysis and Transmission Electron Microscope (TEM). Average zeta size and zeta potential of citrate-capped AuNPs (-vely charged) were found to be 212.7 nm and −38.7 mV respectively {Fig. 1(A,B)}. Polydispersity index (PDI) of AuNPs was found to be 0.366. TEM images showed that AuNPs were spherical in shape, having a size of around 25–30 nm (Fig. 1C). Cytotoxicity assay of citrate-capped AuNPs (-vely charged) was performed in human mammary epithelial (MCF-10A) and triple negative breast cancer (MDA-MB-231 and MDA-MB-468) cells using concentrations ranging from 25 μg/mL to 1 mg/mL for 24 h. MCF-10A cells showed only 30% of cell death even at higher concentration of 1 mg/mL (Fig. 1D). However, significant inhibition in the proliferation of MDA-MB-231 cells was observed upon citrate-capped AuNPs treatment with an IC_50_ value of 720 μg/mL (Fig. 1E). We also observed that citrate-capped AuNPs caused a significant decrease in the proliferation of MDA-MB-468 cells (IC_50_ = 384.35 μg/mL). Data of it was shown in Supplementary Fig. 3A.

**Figure 2:**
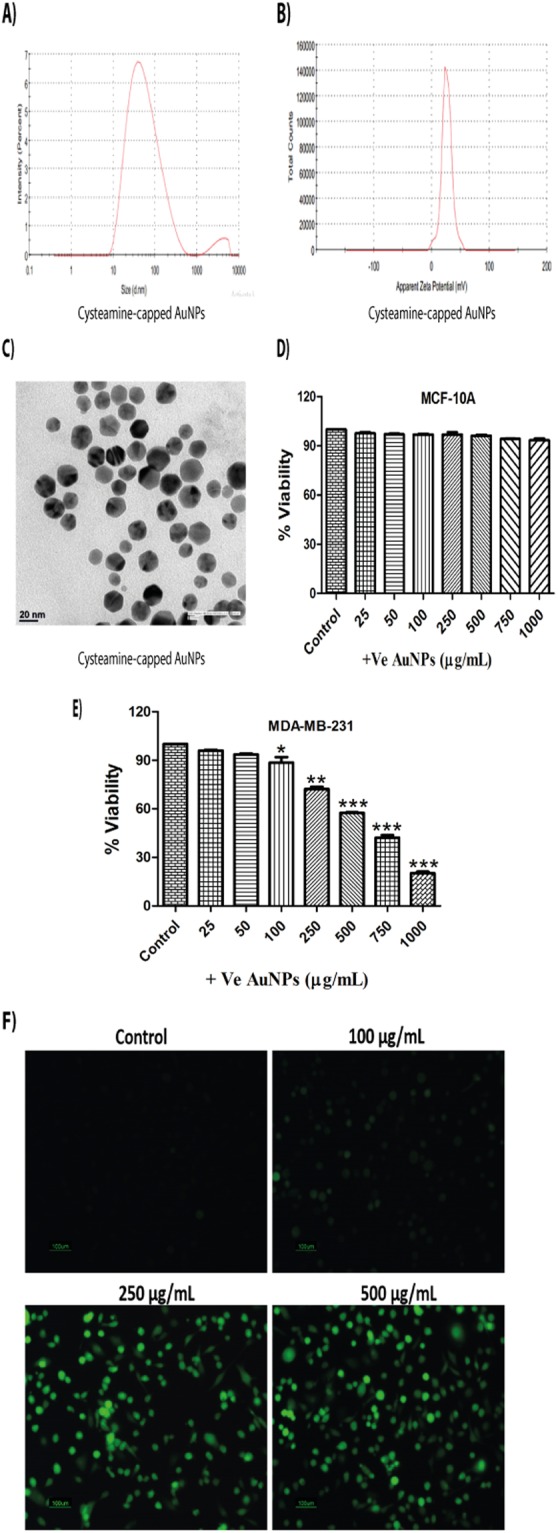
Cysteamine-capped AuNPs exert cytotoxic effects in MDA-MB-231 cells through the induction of oxidative stress. (**A**) Zeta (Average size) of cysteamine-capped AuNPs (**B**) Zeta potential of cysteamine-capped AuNPs (**C**) TEM image of cysteamine-capped AuNPs (**D**) MTT assay of cysteamine-capped AuNPs in MCF-10A cells for 24 h (**E**) MTT assay of cysteamine-capped AuNPs in MDA-MB-231 cells for 24 h (**F**) DCFDA assay of cysteamine-capped AuNPs at concentrations of 100, 250 and 500 μg/mL in MDA-MB-231 cells respectively for 30 min. Results shown are representative of three different experiments. All values were expressed as mean ± S.E.M. *p < 0.05, **p < 0.01, ***p < 0.001 significant vs control.

Characterisation studies on cysteamine-capped AuNPs (+vely charged) revealed 40 nm (average zeta size) and +26.1 mV (zeta potential) {Fig. 2(A,B)}. Polydispersity index (PDI) of AuNPs was found to be 0.4. TEM images show that AuNPs were hexagonal in shape, having a size of around 35–40 nm (Fig. 2C). Cytotoxicity assay revealed that cysteamine-capped AuNPs were less toxic (10% cell death) to human mammary epithelial cells (MCF-10A) even at maximum concentration of 1 mg/mL (Fig. 2D). However, these nanoparticles significantly inhibited the proliferation of MDA-MB-231 cells with much lower IC_50_ value (635 μg/mL) in comparison to −ve charged AuNPs (Fig. 2E). We also observed that cysteamine-capped AuNPs caused a significant decrease in the proliferation of MDA-MB-468 cells (IC_50_ = 313 μg/mL). Data of it was shown in Supplementary Fig. 4A.

Reactive oxygen species (ROS) are highly detrimental to cells and can cause oxidative damage to lipids, proteins and DNA. DCFDA assay was carried out in order to ascertain the role of oxidative stress in cellular damage induced by AuNPs. We observed that 30 min of AuNPs treatment generated oxidative stress in a dose-dependent manner in MDA-MB-231 cells. Oxidative stress generated by cysteamine-capped (+vely charged) AuNPs supports our earlier cell viability data, as they showed more oxidative stress in comparison to citrate-capped AuNPs (Figs 1F and 2F). Oxidative stress was not generated by AuNPs in MCF-10A cells {Supplementary Fig. 1A and B}.

### AuNPs induce cell death by altering Wnt signalling pathway in triple negative breast cancer

**Figure 3:**
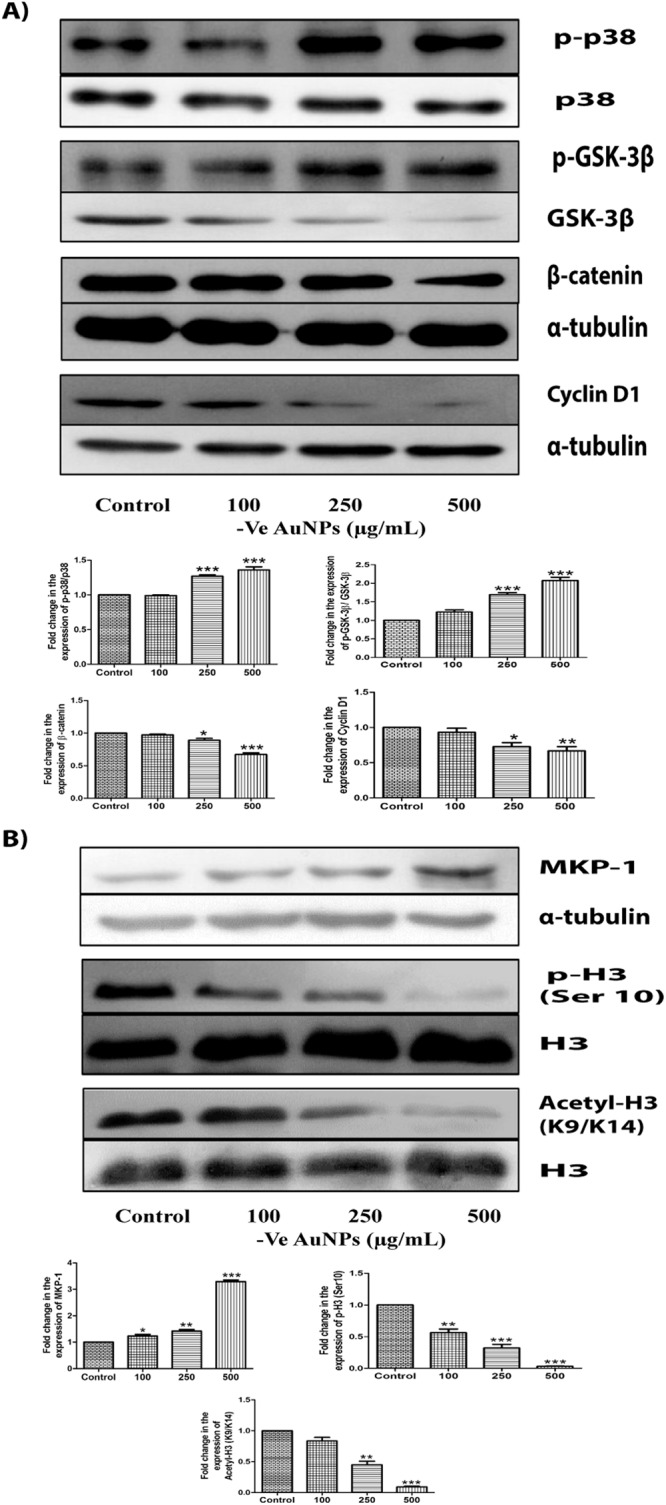
Citrate-capped AuNPs induce cell death by altering Wnt signalling pathway and epigenetic changes in MDA-MB-231 cells. (**A**) Western blots and densitometric analysis of p-p38, p38, p-GSK-3β, GSK-3β, β-catenin and Cyclin D1 when treated with citrate-capped AuNPs of 100 μg/mL, 250 μg/mL and 500 μg/mL for 24 h (**B**) Western blots and densitometric analysis of MKP-1, H3 ser 10 phosphorylation and H3 (K9/K14) acetylation in MDA-MB-231 cells when treated with citrate-capped AuNPs of 100 μg/mL, 250 μg/mL and 500 μg/mL for 24 h. α-tubulin and H3 were used as loading control for our experiments. Results shown were representative of three different experiments. Blots of the same gel have been grouped. After checking the expression of p-p38, p-GSK-3β and histone H3 modifications, stripping was performed and the same blots were reprobed with p38 and GSK-3β and total H3 antibodies. Blots of these were also grouped. Uncropped images of the blots have been shown in supplementary information. All values were expressed as mean ± S.E.M. *p < 0.05, **p < 0.01, ***p < 0.001 significant vs control.

**Figure 4:**
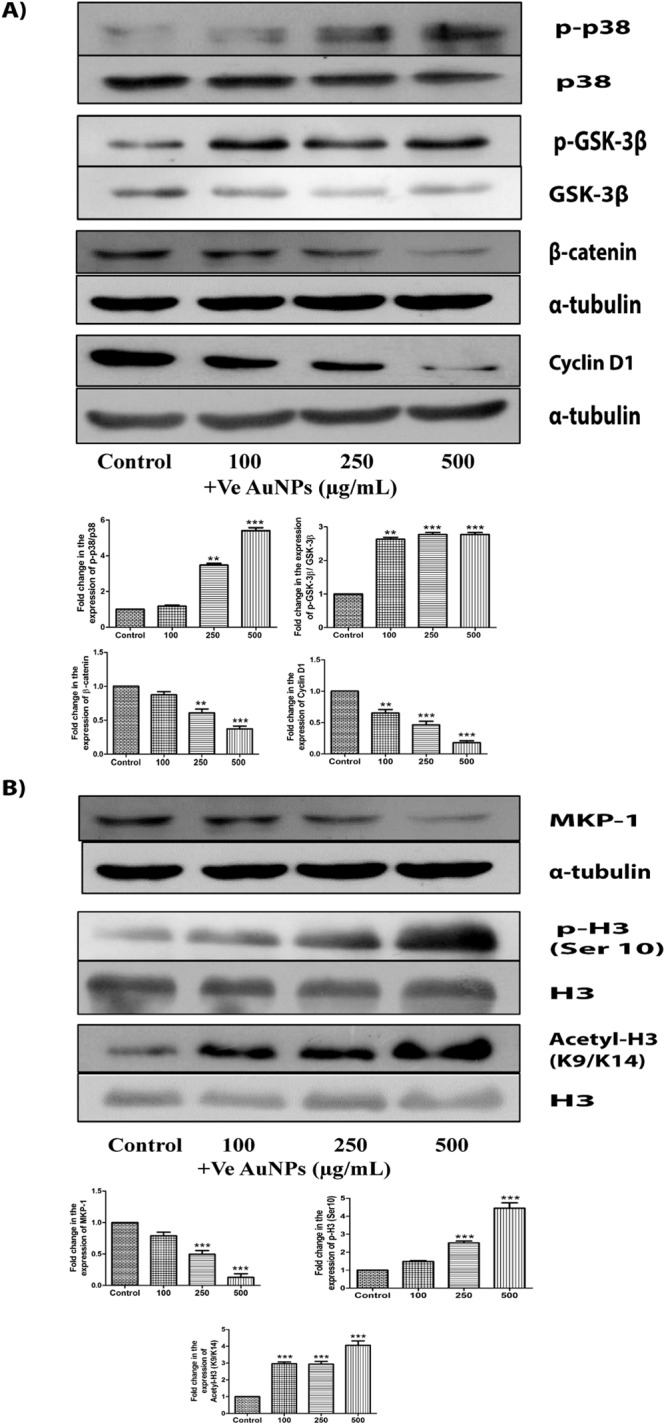
Cysteamine-capped AuNPs induce cell death by altering Wnt signalling pathway and epigenetic changes in MDA-MB-231 cells. (**A**) Western blots and densitometric analysis of p-p38, p38, p-GSK-3β, GSK-3β, β-catenin and Cyclin D1 when treated with cysteamine-capped AuNPs of 100 μg/mL, 250 μg/mL and 500 μg/mL for 24 h (**B**) Western blots and densitometric analysis of MKP-1, H3 ser 10 phosphorylation and H3 (K9/K14) acetylation in MDA-MB-231 cells when treated with cysteamine-capped AuNPs of 100 μg/mL, 250 μg/mL and 500 μg/mL for 24 h. α-tubulin and H3 were used as loading control for our experiments. Results shown were representative of three different experiments. Blots of the same gel have been grouped. After checking the expression of p-p38, p-GSK-3β and histone H3 modifications, stripping was performed and the same blots were reprobed with p38, GSK-3β and total H3 antibodies. Blots of these were also grouped. Uncropped images of the blots have been shown in supplementary information. All values were expressed as mean ± S.E.M. **p < 0.01, ***p < 0.001 significant vs control.

AuNPs induce oxidative stress and thereby cell death in variety of cancers. Oxidative stress induces phosphorylation of p38 and contributes to apoptosis in many cancers. We also noticed significant increase in phosphorylated p38 levels upon AuNPs treatment (Figs 3A and 4A). Phosphorylated p38 phosphorylates GSK-3β and reduce its activity. Phosphorylation of GSK-3β by p38 MAP kinase can also destabilise β-catenin and inhibits the transcription of target genes like Cyclin D1. The major components involved in Wnt signaling pathway are GSK-3β, β-catenin and Cyclin D1. We also observed that AuNPs phosphorylated GSK-3β, and decreased the expression of total GSK-3β. Inactivation of GSK-3β by phosphorylation also reduced the expressions of β-catenin and Cyclin-D1 in a dose-dependent manner (Figs 3A and 4A). SIRT1 deacetylates β-catenin and promotes the proliferation of cells in various cancers. AuNPs significantly reduced the expression of SIRT1 in MDA-MB-231 cells {Supplementary Fig. 2A and B}. We also observed that AuNPs decreased the expression of GSK-3β and β-catenin in MDA-MB-468 cells (Supplementary Figs 3B and 4B). Comparing +vely and −vely charged AuNPs, we observed that alteration in Wnt signalling pathway is more predominant with +vely charged AuNPs in MDA-MB-231 cells.

### Epigenetic alterations by AuNPs in triple negative breast cancer

AuNPs-induced oxidative stress can either cause the activation of MAP kinases/ MAPK phosphatases (MKP-1) or even both. AuNPs increased the activation of MAP kinases (p38) in MDA-MB-231 cells. Since MKP-1 plays a key role in the deactivation of various proteins and also gets altered by MAP kinases, we checked the protein expression of MKP-1 and observed a significant increase in its levels with citrate-capped AuNPs (−vely charged) in MDA-MB-231 and MDA-MB-468 cells (Fig. 3B and Supplementary Fig. 3B). However, cysteamine–capped AuNPs (+vely charged) significantly decreased MKP-1 expression levels (Fig. 4B and Supplementary Fig. 4B). The difference in the expression of MKP-1 with the charge of AuNPs suggested us to look for its downstream targets.

MKP-1 is involved in maintaining the phosphorylation levels of histone H3 and various other proteins. As histone H3 serine 10 phosphorylation plays a crucial role in the progression and arrest of cell cycle, we also decided to check the phosphorylation of histone H3 at Ser 10. We observed a significant decrease in its expression levels with citrate-capped AuNPs (−vely charged) in a dose-dependent manner in MDA-MB-231 and MDA-MB-468 cells (Fig. 3B and Supplementary Fig. 3B). However, cysteamine-capped AuNPs (+vely charged) increased the histone H3 Ser 10 phosphorylation in a dose-dependent manner in MDA-MB-231 and MDA-MB-468 cells (Fig. 4B and Supplementary Fig. 4B). Acetylation on lysine 9 at histone H3 has been reported to play an important role in the alteration of histone H3 modifications on serine 10. So, we checked whether acetylation of H3 (K9/K14) is also involved in AuNPs-induced cell death of MDA-MB-231 cells. Deacetylation of H3 at (K9/K14) was observed with citrate-capped AuNPs (−vely charged) (Fig. 3B). However, cysteamine-capped AuNPs (+vely charged) acetylated histone H3 at (K9/K14) in MDA-MB-231 cells (Fig. 4B).

### Electron micrographs showing the distribution and internalisation of AuNPs in MDA-MB-231 cells

**Figure 5:**
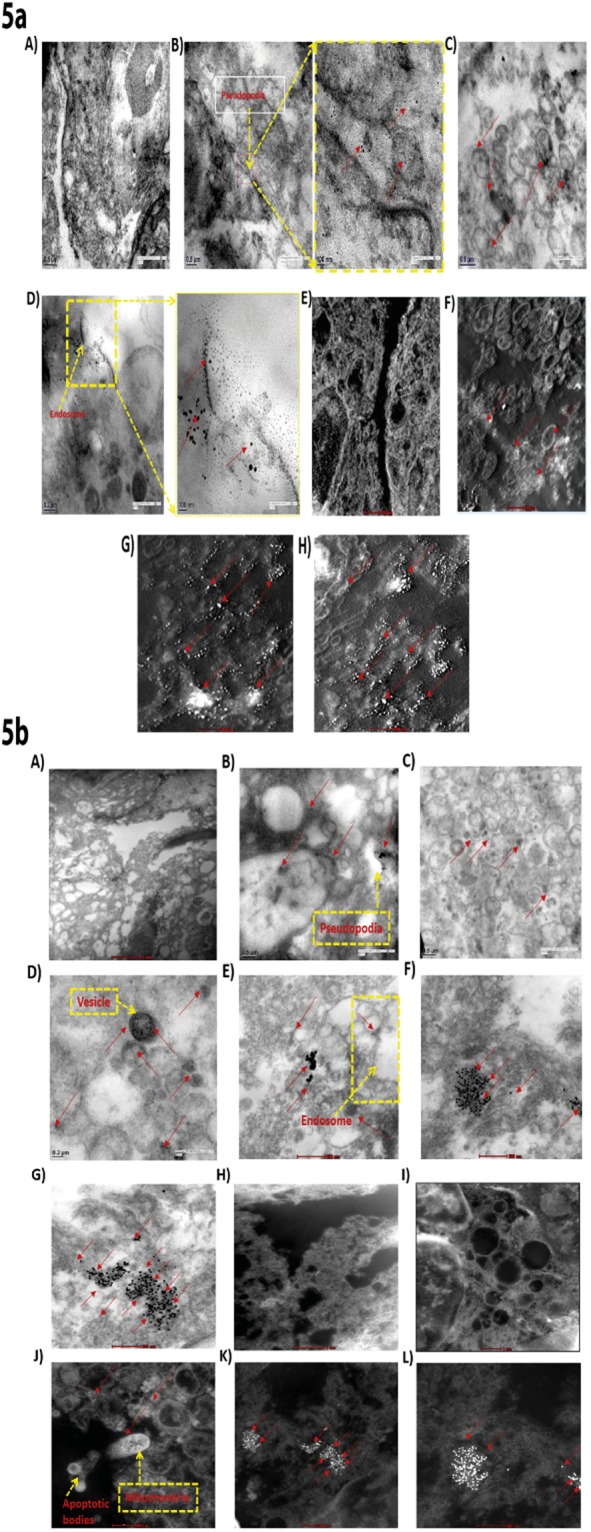
Electron micrographs showing the distribution and internalisation of AuNPs in MDA-MB-231 cells. 5a (A) & 5b (A) show TEM images of untreated MDA-MB-231 cells 5a (B) and 5b (B) Formation of pseudopodia by MDA-MB-231 cells with AuNPs in centre; 5a (C) & 5b (C,D) Localisation of AuNPs inside the vesicles; 5a (D) & 5b (E) Internalisation of AuNPs; 5a (E) and 5b (H,I) HRTEM images of untreated MDA-MB-231 cells; 5a (F–H) HRTEM images of MDA-MB-231 cells treated with 100 μg/mL, 250 μg/mL and 500 μg/mL of citrate-capped AuNPs respectively; 5b (F,G) TEM images of cysteamine-capped AuNPs treated cells; 5b (J) HRTEM image showing localisation of cysteamine-capped AuNPs inside the mitochondria; 5b (K,L) HRTEM image of MDA-MB-231 cells treated with cysteamine-capped AuNPs. Red arrows in the images are pointing towards AuNPs.

AuNPs treated cell sections were stained and observed under TEM to see the distribution of AuNPs within MDA-MB-231 cells as described in experimental procedures (Supplementary information). We noticed from Fig. 5a(B) and 5b(B) that, cells were protruding their pseudopodia for the uptake of AuNPs. Moreover, AuNPs were found to be entrapped inside the vesicles of organelles {Fig. 5a(C) and 5b(C,D)}. Citrate-capped AuNPs (−vely charged) were found to be localised more in cytoplasm in comparison to cysteamine-capped AuNPs (+vely charged). On the other hand, HRTEM images revealed that cysteamine-capped AuNPs entered inside the mitochondria and are forming apoptotic bodies {Fig. 5b(J)}. Formation of the endosome containing large number of AuNPs near the cell membrane was also observed in AuNPs treated cells, suggesting that endocytosis is involved in AuNPs uptake {Fig. 5a(D) and b(E)}. Moreover, electron micrographs show that AuNPs were causing damage to the cells monolayer, as visible by the expulsion of fluids from inside of the organelles {Fig. 5a(F–H), 5b(F,G) and 5b(K,L)}.

### AuNPs makes MDA-MB-231 cells sensitive to 5-Fluorouracil (5-FU) by decreasing thymidylate synthetase expression

**Figure 6:**
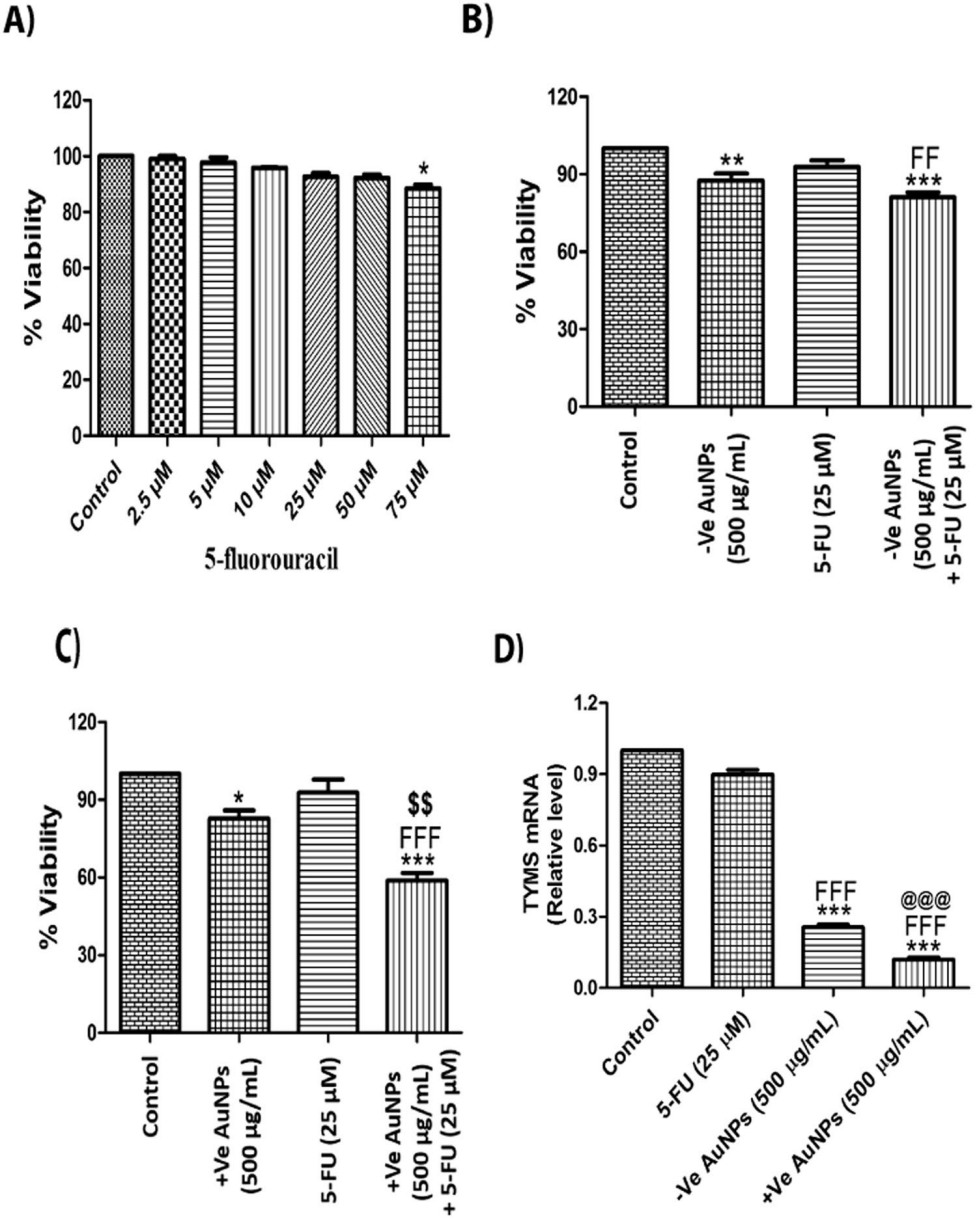
AuNPs makes MDA-MB-231 cells sensitive to 5-Fluorouracil by decreasing thymidylate synthetase expression. (**A**) MTT assay of 5-Fluorouracil in MDA-MB-231 cells for 24 h (**B**) MTT assay with citrate-capped AuNPs pre-treatment (500 µg/mL) for 12 h and 5-FU post-treatment (25 µM) for 12 h in MDA-MB-231 cells (**C**) MTT assay with cysteamine-capped AuNPs pre-treatment (500 µg/mL) for 12 h and 5-FU post treatment (25 µM) for 12 h in MDA-MB-231 cells (**D**) Quantitative RT-PCR of TYMS upon AuNPs (500 µg/mL) and 5-FU treatment (25 µM) for 12 h in MDA-MB-231 cells. Results shown are representative of three different experiments. All values were expressed as mean ± S.E.M. *p < 0.05, ***p < 0.001 significant vs control; ^FFF^p < 0.001 vs 5-FU; ^@@@^p < 0.001 vs AuNPs (−ve); ^$$^p < 0.01 vs AuNPs (+ve).

Present neoadjuvant chemotherapies for TNBC include the combinations of anti-cancer drugs like 5-FU, doxorubicin/epirubicin and cyclophosphamide (FAC or FEC) or paclitaxel/docetaxel followed by 5-FU, doxorubicin/epirubicin and cyclophosphamide (TFAC or TFEC). However, even with chemotherapy, patients with TNBC have worse outcomes when compared with other breast cancer subtypes because of the inherent adverse prognostic factors associated with the disease. Herein, cell viability assay was performed on 5-FU in MDA-MB-231 cells at doses ranging from 2.5 μM to 75 μM for 24 h. 5-FU showed only 12% decrease in cell growth even at 75 μM concentration (Fig. 6A). We chose 25 μM of 5-FU for sensitivity study in order to check whether the AuNPs pre-treatment (500 μg/mL) for 12 h could sensitize the MDA-MB-231 cells to 5-FU at dose of 25 μM, which is initially not effective in triple negative breast cancer. Citrate-capped AuNPs (−vely charged) alone caused 13% cell death in MDA-MB-231 cells when treated for 12 h, but pre-treatment of AuNPs for 12 h sensitized the MDA-MB-231 cells to 5-FU and showed around 19% cell death (Fig. 6B). However, cysteamine-capped AuNPs (+vely charged) alone caused 18% cell death in MDA-MB-231 cells when treated for 12 h (Fig. 6C). Interestingly, cysteamine-capped AuNPs pre-treatment for 12 h sensitized the MDA-MB-231 cells to 5-FU and showed 42% cell death. MDA-MB-231 cells pre-treated with +vely charged AuNPs showed almost 5-fold increase in the sensitivity to 5-FU. Thymidylate synthetase (TYMS) overexpression is one of the major factors involved in 5-FU drug resistance. Herein, we have checked the effect of AuNPs and 5-FU on TYMS mRNA expression. Our data shows that AuNPs treatment for 12 h drastically decreased the expression of thymidylate synthetase (Fig. 6D).

## Discussion

In the present study, differentially charged AuNPs were synthesized as described in the experimental procedures (Supplementary information). Characterisation studies by zeta size, zeta potential analysis and TEM, revealed that, both citrate-capped (−vely charged) and cysteamine-capped (+vely charged) AuNPs were in the size range of 25–40 nm, uniformly dispersed and stable. Cytotoxicity assay showed a significant decrease in the proliferation of MDA-MB-231 and MDA-MB-468 cells in a dose-dependent manner whereas, normal breast epithelial cells (MCF-10A) showed little toxicity to AuNPs. Multiple studies have shown that AuNPs exert their cytotoxicity through the induction of oxidative stress. In HeLa cells, AuNPs induced oxidative stress, leading to protein and lipid oxidation, severely impaired mitochondrial function, and eventually cell death^23^. We also observed that AuNPs-induced oxidative stress in MDA-MB-231 cells but not in MCF-10A cells, suggesting that AuNPs induced-oxidative stress is responsible for the cell death of MDA-MB-231 cells.

Several reports suggest that oxidative stress causes the activation of p38 MAPK pathway^15^. Herein, we also observed that AuNPs increased the phosphorylation of p38 MAP Kinases in MDA-MB-231 cells. Accumulating evidence suggests that phosphorylated p38 causes phosphorylation of GSK-3β and makes it inactive^24^. We also found that AuNPs increased the phosphorylation of GSK-3β and decreased the total expression of GSK-3β in MDA-MB-231 and MDA-MB-468 cells. In the absence of canonical Wnt signaling, phosphorylation of β-catenin initially occurs in a complex containing APC (adenomatous polyposis coli) and AXIN, a scaffolding protein by casein kinase Iα (CKIα) at serine 45, which facilitates glycogen synthase kinase 3β (GSK-3β)- to phosphorylate serine/threonine residues in the positions of 41, 37 and 33^25^. A wealth of data has revealed that SIRT1 deacetylates β-catenin and promotes the nuclear translocation of β-catenin and thereby causing the proliferation of cells in various cancers^26,27^. β-catenin translocates to the nucleus and binds to TCF/LEF family of transcription factors and enhance the transcription of various genes like cyclin D1 and Myc^28^. In our study, we also observed that β-catenin, Cyclin D1 and SIRT1 protein expressions were reduced upon treatment of AuNPs in a dose- dependent manner in MDA-MB-231 and MDA-MB-468 cells, suggesting that AuNPs induce alterations in the Wnt signalling pathway, which contributes to cell death and SIRT1 might be also involved in the proliferation of MDA-MB-231 cells through regulation of β-catenin. In addition, genome-wide mRNA expression analysis demonstrated that treatment with AuNPs caused up-regulation of stress-related and inflammation-related genes and a concomitant decrease in the expression of cell cycle genes^29^. Wnt signalling regulates proliferation, survival and differentiation, and thereby plays a key role in embryonic development and tumorigenesis^30^. Altering Wnt signalling seems to be effective option in the treatment of TNBC^31–34^.

Mitogen-activated protein kinase phosphatase-1 (MKP-1) can also get activated by oxidative stress to regulate the phosphorylation of various other proteins. Our data shows that −vely charged AuNPs increased MKP-1 protein expression, whereas +vely charged AuNPs decreased it in MDA-MB-231 and MDA-MB-468 cells. In line with our results, Schaeublin *et al*. also observed that altering the surface charge of gold nanoparticles induced different biological responses in a human keratinocyte cell line^35^.

Altered responses of MKP-1 to differentially charged AuNPs made us to look for change in the levels of histone phosphorylation. As MKP-1 is involved in maintaining the phosphorylation levels of histone H3, we checked the effect of AuNPs on histone H3 ser10 phosphorylation. Phosphorylation of histone H3 plays an important role in the progression and arrest of cell cycle. Particularly, phosphorylation of histone H3 at serine 10 has been shown to be associated with chromosome condensation prior to mitosis^36^. Apart from its involvement in chromosome condensation, phosphorylation of histone H3 on serine 10 is also induced by various death stimuli, indicating its role in apoptosis and cell death^37,38^. Phosphorylation of histone H3 at ser10 appears in the G2/M phase and is used as cell cycle marker to index the G2/M stages. Increased phosphorylation of histone H3 at serine 10 is also associated with premature mitosis in p53 negative cells depleted of checkpoint kinase 1 (CHK1)^39^. We observed a significant dephosphorylation of histone H3 at Ser 10 on treatment with −vely charged AuNPs in MDA-MB-231 and MDA-MB-468 cells, indicating that these cells cannot undergo chromosome condensation and fail to enter mitosis. However, +vely charged AuNPs increased the serine 10 phosphorylation of histone H3 in MDA-MB-231 and MDA-MB-468 cells, suggesting that these cells are undergoing mitotic catastrophy and cell death. Moreover, the level of epigenetic alterations was more in MDA-MB-231 cells as compared to MDA-MB-468 cells by differentially charged AuNPs. The possible reason might be due to the differences in the level of metastasis in the cells. Interaction of cysteamine-capped (+vely charged) AuNPs with DNA initiates untimely and abrupt chromatin folding, resulting into mitotic catastrophy. This is well supported by our TEM data, which revealed that +vely charged AuNPs disrupts cells monolayer completely, resulting into sudden death of MDA-MB-231 cells. TEM images further revealed that +vely charged AuNPs were localised in mitochondria, which might result in loss of mitochondrial membrane potential, leading to mitochondrial dysfunction and eventually cell death. However, −vely charged AuNPs are only localised in cytosol and induce cell death at much slower rate. Different endocytotic uptake mechanisms are responsible for intra-vesicular particle localization, such as clathrin or caveolin-mediated endocytosis^22^. TEM images also revealed that AuNPs enter inside the MDA-MB-231 cells through endocytosis. Various reports have demonstrated the associations of histone H3 lysine 9/lysine 14 acetylation and histone H3 serine10 phosphorylation^40,41^. It is earlier reported that HDAC inhibitors like suberoylanilide hydroxamic acid (SAHA) increased the protein expression levels of histone H3 (K9/K14) acetylation on the promoter region of p21 gene and thereby supressed cancer cell growth in multiple myeloma^42^. Our data shows that +vely charged AuNPs promotes H3 (K9/K14) acetylation in line with the serine 10 phosphorylation, whereas −vely charged AuNPs prevents ser 10 H3 phosphorylation and H3 (K9/K14) acetylation.

5-Fluorouracil (5-FU) is widely used for the treatment of TNBC but drug resistance and sensitivity of the cells represent a major clinical challenge over the course of therapy. Multiple factors are involved in 5-FU resistance like high level expression of thymidylate synthetase and overexpression of Bcl-2, BCL-XL and Mcl-1 proteins^43^. We provide evidence that +vely charged AuNPs pre-treatment significantly sensitized MDA-MB-231 cells to 5-FU in comparison to –vely charged AuNPs. Furthermore, pre-treatment of +vely charged AuNPs drastically reduced the mRNA levels of thymidylate synthetase in comparison to –vely charged AuNPs. This explains the increased sensitivity of MDA-MB-231 cells to 5-FU by +vely charged AuNPs. Further studies are in progress to check whether alteration in expression of thymidylate synthetase by AuNPs is epigenetically regulated. Understanding of which will be of profound clinical significance.

## Conclusion

Differentially charged gold nanoparticles (AuNPs)-induced cytotoxicity in TNBC cells involves generation of oxidative stress, alteration of Wnt signalling pathway and histone H3 modifications at serine 10 and lysine (9/14) residues respectively. The surface potential of AuNPs alters different epigenetic modifications. Negatively charged AuNPs induced slow process of cell death whereas, positively charged AuNPs caused abrupt destruction of MDA-MB-231 cells due to increased histone H3 ser 10 phosphorylation (mitotic catastrophy). Cell type dependent epigenetic alterations were observed with differentially charged AuNPs. Additionally, we report for the first time that treatment of AuNPs makes MDA-MB-231 cells sensitive to 5-FU by decreasing thymidylate synthetase expression. We propose that epigenetic changes caused by AuNPs might be responsible for making MDA-MB-231 cells sensitive to 5-FU. Further studies are required to warrant any conclusion.

## Electronic supplementary material

Supplementary Information

## Data Availability

All data generated or analysed during this study are included in this published article (and its Supplementary information files).
