# Supplementary material for: Gold nanoparticles-induced cytotoxicity in triple negative breast cancer involves different epigenetic alterations depending upon the surface charge
Source: Sci Rep. 2018 Aug 16;8:12295. doi: 10.1038/s41598-018-30541-3 (PMC6095919; doi:10.1038/s41598-018-30541-3)
Supplement: Supplementary file 1 — Supplementary Information [file 41598_2018_30541_MOESM1_ESM.pdf]

Gold nanoparticles-induced cytotoxicity in triple negative breast cancer involves different epigenetic alterations depending upon the surface charge

Sunil Kumar Surapaneni[a], Shafiya Bashir[b], Kulbhushan Tikoo[*]

List of Materials included

1) Experimental procedures

2) Supplementary figures

3) Uncropped blots images

4) References

1) Experimental procedures

a) Synthesis of citrate-capped AuNPs

Citrate-capped AuNPs were prepared by the reduction of Gold (III) chloride trihydrate ($HAuCl_4.3H_2O$) with trisodium citrate according to the Turkevich and Frens method [1,2]. Briefly, the reaction was carried out in a 250 mL round bottomed flask with the centre neck attached to a reflux condenser. First, 250 mL of 0.25 mM $HAuCl_4.3H_2O$ solution was heated to boiling. Then, 4 mL of aqueous solution of 1% trisodium citrate was added to it under vigorous stirring and the boiling was continued for another 15 min until the solution turns to a deep red colour. During the reaction, the colour of the solution changed initially from yellow to colourless and finally to wine red. It was then stirred until it reached room temperature to control the particle size and thus achieving a narrow particle size distribution.

b) Synthesis of cysteamine-capped AuNPs

Cysteamine-capped gold nanoparticles were prepared by the reduction of gold (III) chloride trihydrate with sodium borohydride in the presence of cysteamine [3]. Briefly, 400 μL of 213 mM cysteamine hydrochloride was added to 40 mL of 1.42 mM gold (III) chloride trihydrate in a conical flask and then subjected to stirring for 20 min at room temperature. Sodium borohydride was dissolved in cold distilled water immediately before use. After 20 min, 10 μL of 10 mM $NaBH_4$ was added quickly into the mixture solution. Vigorous stirring was maintained from the addition of $NaBH_4$ to another 30 min. The colour of mixture solution during the reaction process was changed from yellow to brownish. After further mild stirring, the gold nanoparticle solution was stored in the dark condition at 4 °C.

c) Zeta size, Zeta potential and TEM

Dynamic light scattering for characterization of hydrodynamic size of AuNPs dispersed in water was performed on Nano-ZS, Malvern Instruments, Malvern, UK, taking the average of 5 measurements. Zeta potential was also measured to determine the amount of aggregation of particles [4]. AuNPs were analyzed under TEM (FEI TF-20) for size determination. Briefly, the stock solution of AuNPs synthesized by the above method was diluted in water followed by sonication. 2 drops of the solution were dropped onto carbon coated copper grids (from Ted Pella INC) and kept for drying. The dried sample was analyzed under the microscope at 200 kV.

d) Transmission electron microscopy (TEM) of AuNPs treated cells

Ultra-thin sections of cells were analyzed using TEM to see the distribution of AuNPs according to the modified method as described [5]. Briefly, cells were treated with AuNPs (100 μg/mL, 250 μg/mL and 500 μg/mL) for 24 h. At the end of incubation, the cells were washed with PBS to remove any unbound AuNPs. Cells were then fixed in 2.5% glutaraldehyde for 30 min at 4 °C. Fixed cells were scraped and washed 5 times with PBS. Before dehydration with

increasing concentrations of alcohol, cells were further treated with 1 % osmium tetroxide for 2 h at 4 °C. Alcohol and spurr's low viscosity resin were used in the ratio 2:1, 1:1, 1:2 and 1:3. Finally 100 % spurr resin was added and the beam capsule was incubated for 18 h at 70 °C. RMC ultra-microtome is used for cutting ultra-thin sections of 60 nm thickness. Sections were stained with 0.5% uranyl acetate and analyzed under FEI TF-20 TEM at 120 kV.

e) Isolation of total proteins and western blotting

After AuNPs treatment for 24 h, isolation of total proteins and histones, using 0.25 M HCl were done by the methods as described [6]. Protein estimation was performed by Lowry's method and then reduced by using 1X laemmli's sample buffer. Equal amounts were run on SDS PAGE and were electrophoretically transferred onto PVDF membrane using semi-dry transfer apparatus (Bio-Rad). Immunoblot analysis was performed using anti-phospho-p38 (rabbit,1:1000, Santa Cruz Biotechnology, CA, USA), anti-p38 (rabbit,1:1000, Santa Cruz Biotechnology, CA, USA), anti-phospho-GSK-3β (rabbit,1:1000, Santa Cruz Biotechnology, CA, USA), anti-GSK-3β (rabbit,1:1000, Santa Cruz Biotechnology, CA, USA), anti-β-catenin (rabbit,1:1000, Santa Cruz Biotechnology, CA, USA), anti-CyclinD1 (rabbit,1:500, Santa Cruz Biotechnology, CA, USA), anti-MKP-1 (rabbit,1:500, Santa Cruz Biotechnology, CA, USA), anti-histone H3 ser 10 phosphorylation (rabbit,1:500, Cell signaling technology, USA), anti-acetyl H3 (K9/K14) (rabbit,1:500, Cell signaling technology, USA), anti-H3 (goat,1:1000 Sigma, St. Louis, MO, USA), and anti α-tubulin (mouse, 1:1000, Santa Cruz Biotechnology, CA, USA) primary antibodies. The antigen-antibody complex was detected using HRP-coupled secondary antibodies (Santa Cruz Biotechnology, CA, USA). Specific bands were detected by chemiluminescence (NOVEX Invitrogen), and visualization was performed by exposure of the membranes to hyperfilm (GE Healthcare). For subsequent antibody treatments, the membranes were stripped in stripping buffer and re-probed with another antibody. The immunoblots were quantified by densitometry scanning using NIH Image J software.

f) Total RNA isolation

Briefly, total RNA was extracted from cells using TRIzol reagent (Invitrogen, CA, USA) and was purified according to the manufacturer's protocol using an RNeasy kit (AuPrep RNeasy Mini Kit; Life Technologies Pvt. Ltd., India) as described [7]. The RNA quality and integrity of each sample was assured using NanoDrop spectrophotometer (ND-1000) by A260/280 absorbance ratio and agarose gel electrophoresis respectively.

g) Reverse transcription and RT-PCR

For checking the mRNA levels of genes, cDNA synthesis from RNA was carried out by using verso cDNA synthesis kit (Thermo Fisher Scientific, USA) according to the manufacturer's instructions as described [7] and then Quantitative real time-PCR was carried out by using Light Cycler 2.0 (Roche Diagnostics) according to the method as described [6]. Specific primers for thymidylate synthetase, TYMS (Fwd:5'-TCTGCTGACAACCAAACGTG-3'and Rev:5'-GGATTCCAAGCGCACATGAT-3') were purchased from Eurofins Genomics India Pvt. Ltd. (Bangalore, India). After amplification, a melt curve analysis was performed to verify the specificity of the reaction. The analysis was performed using Light Cycler software (Roche Diagnostics, USA). Relative gene expression was assessed using the comparative Ct (ΔCt) method and was normalized to 18S rRNA (Fwd:5'-GCAATTATTCCCCATGAACG-3' and Rev: 5'-AGGGCCTCACTAAACCATCC-3').

2) Supplementary Figures:

Figure 1

A)

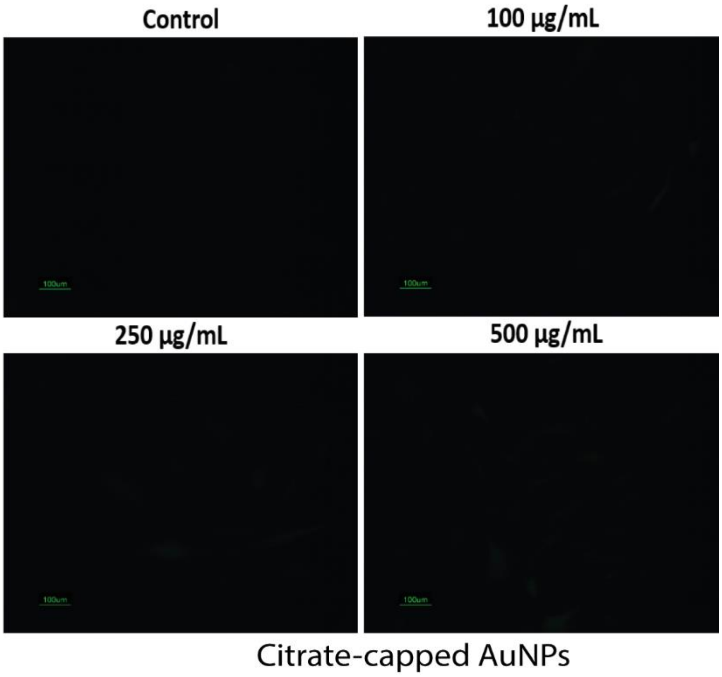

B)

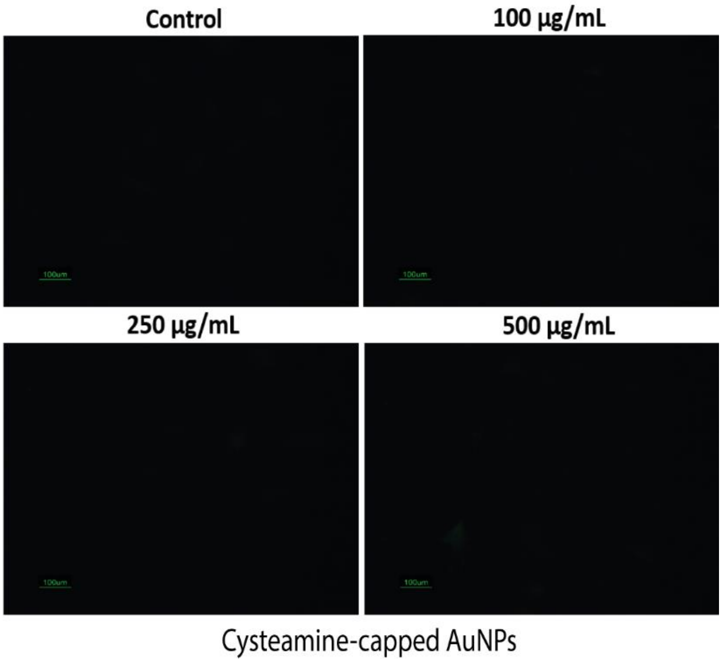

Supplementary Figure 1: Effect of Gold nanoparticles on the oxidative stress in MCF-10A cells

A) DCFDA assay of citrate-capped AuNPs at concentrations of 100, 250 and 500 µg/mL in MCF-10A cells for 30 min B) DCFDA assay of cysteamine-capped AuNPs at concentrations of 100, 250 and 500 µg/mL in MCF-10A cells for 30 min. Untreated cells were considered as the control group. Results shown are representative of three different experiments. Uncropped images of these blots were also shown in supplementary information.

Figure 2

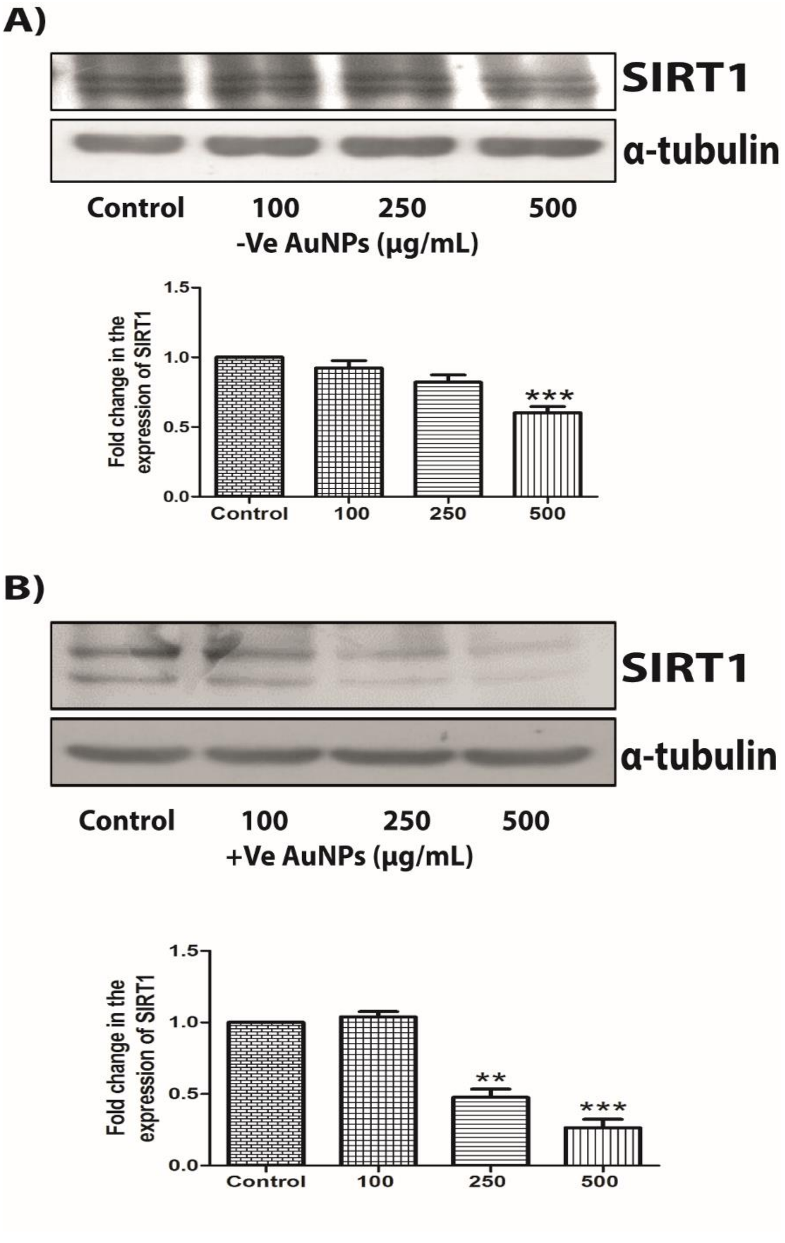

Supplementary Figure 2: Western blot and densitometric analysis of SIRT1 in MDA-MB-231 cells when treated with citrate- and cysteamine-capped AuNPs of 100 µg/mL, 250 µg/mL and 500 µg/mL for 24 h. α-tubulin was used as loading control for our experiments. Results shown were representative of three different experiments. All values were expressed as mean ± S.E.M. **p<0.01, ***p<0.001 significant vs control

Figure 3: Effect of citrate-capped AuNPs in MDA-MB-468 cells

A)

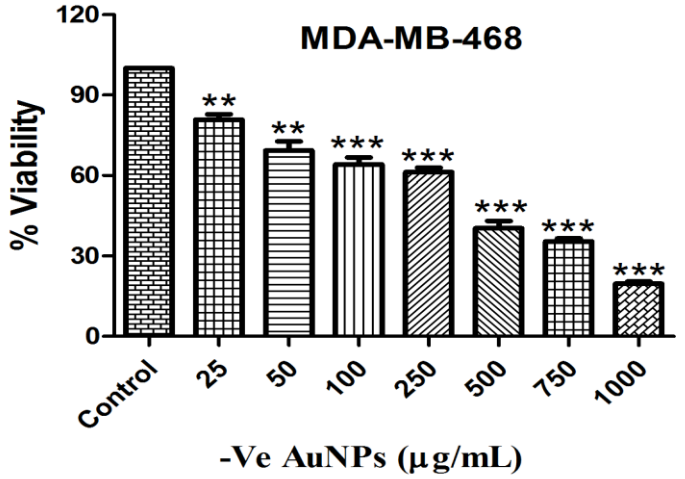

B)

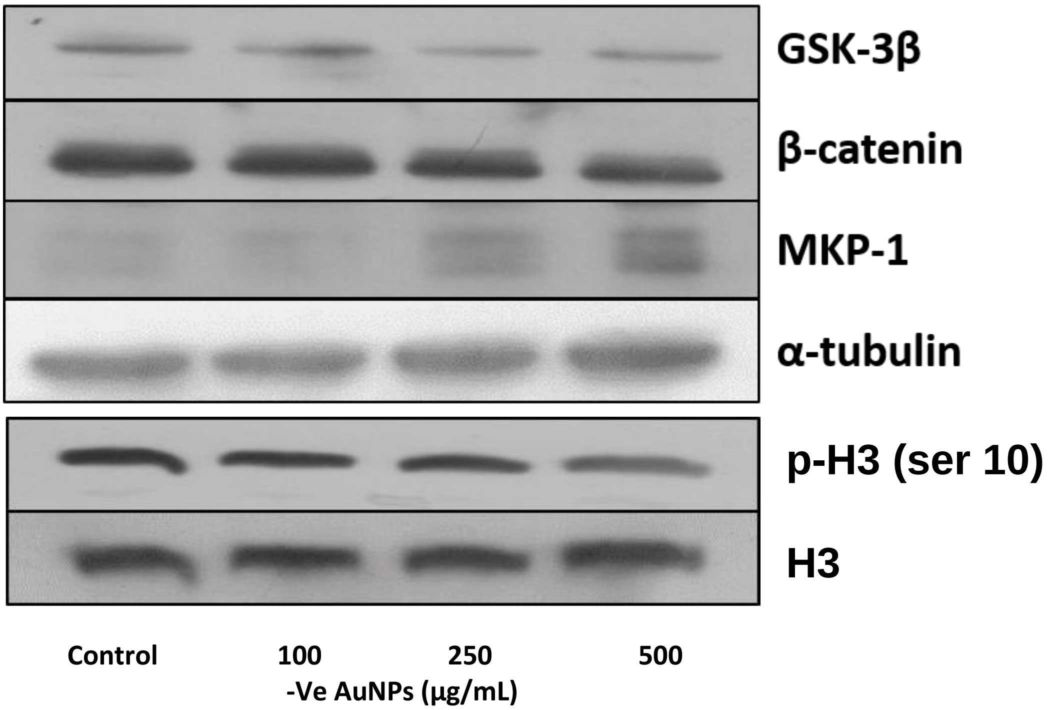

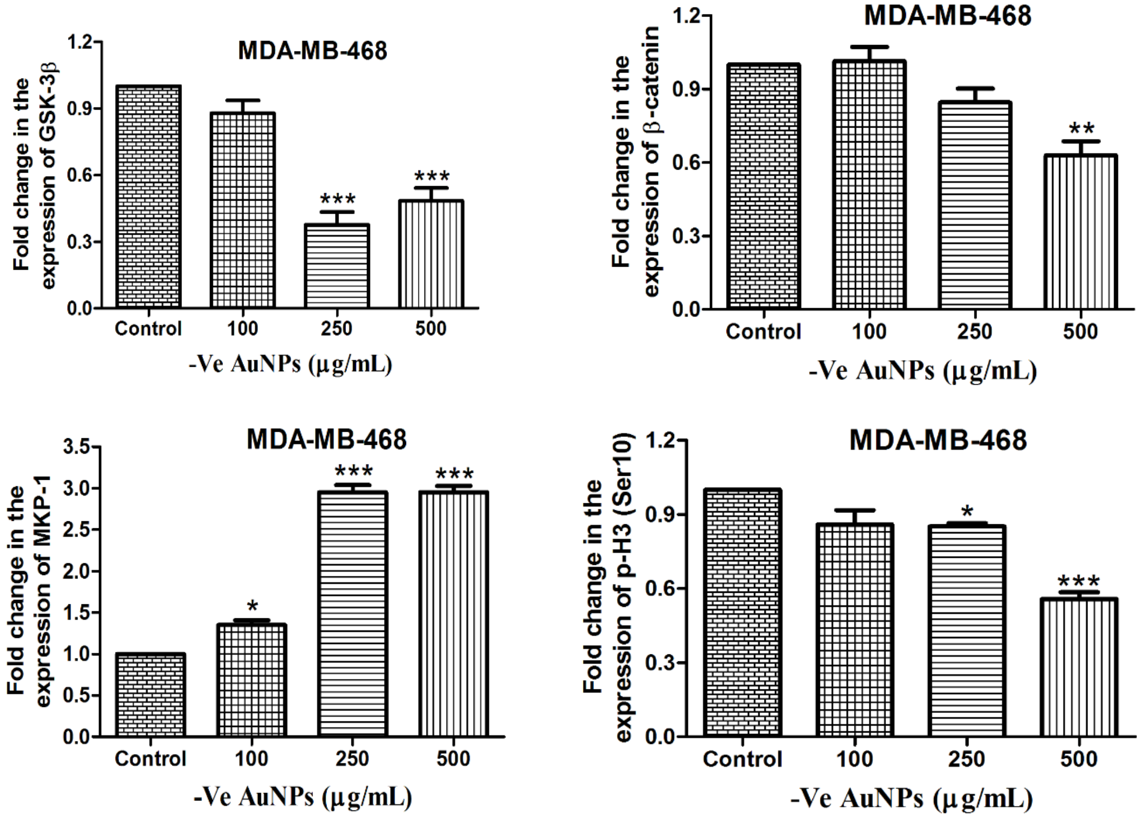

Supplementary Figure 3: A) MTT assay of citrate-capped AuNPs in MDA-MB-468 cells for 24 h B) Western blots and densitometric analysis of GSK-3β, β-catenin, MKP-1 and H3 ser 10 phosphorylation in MDA-MB-468 cells when treated with citrate-capped AuNPs of 100 μg/mL, 250 μg/mL and 500 μg/mL for 24 h. α-tubulin and H3 were used as loading control for our experiments. Results shown were representative of three different experiments. All values were expressed as mean ± S.E.M. *$p<0.05$, **$p<0.01$, ***$p<0.001$ significant vs control

Figure 4: Effect of cysteamine-capped AuNPs in MDA-MB-468 cells

A)

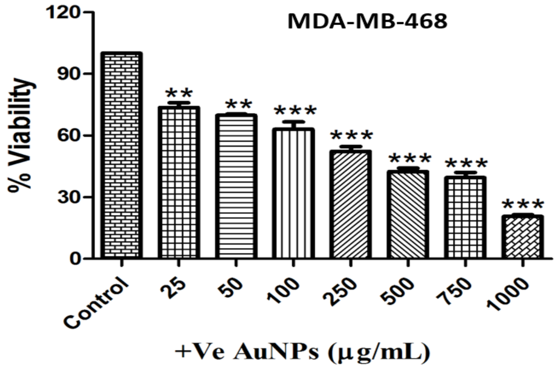

B)

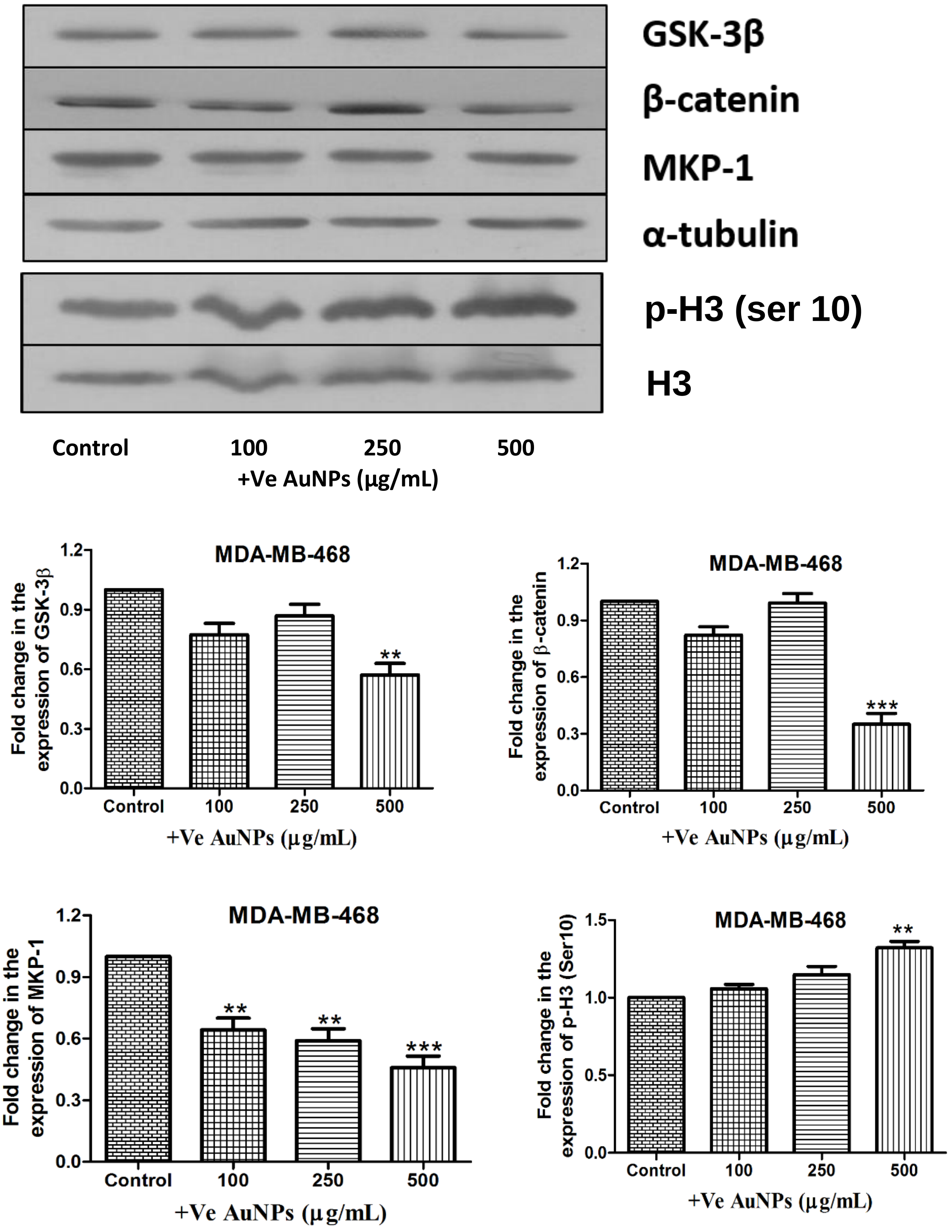
GSK-3β
β-catenin
MKP-1
α-tubulin
p-H3 (ser 10)
H3
Control
100
250
500
+Ve AuNPs (μg/mL)
MDA-MB-468
Fold change in the expression of GSK-3β
1.2
0.9
0.6
0.3
0.0
**
Control
100
250
500
+Ve AuNPs (μg/mL)
MDA-MB-468
Fold change in the expression of β-catenin
1.2
0.9
0.6
0.3
0.0
***
Control
100
250
500
+Ve AuNPs (μg/mL)
MDA-MB-468
Fold change in the expression of MKP-1
1.2
0.9
0.6
0.3
0.0
**
**
***
Control
100
250
500
+Ve AuNPs (μg/mL)
MDA-MB-468
Fold change in the expression of p-H3 (Ser10)
1.5
1.0
0.5
0.0
**
Control
100
250
500
+Ve AuNPs (μg/mL)

Supplementary Figure 4: A) MTT assay of cysteamine-capped AuNPs in MDA-MB-468 cells for 24 h B) Western blots and densitometric analysis of GSK-3β, β-catenin, MKP-1 and H3 ser 10 phosphorylation in MDA-MB-468 cells when treated with cysteamine-capped AuNPs of 100 μg/mL, 250 μg/mL and 500 μg/mL for 24 h. α-tubulin and H3 were used as loading control for our experiments. Results shown were representative of three different experiments. All values were expressed as mean ± S.E.M. $^{**}p<0.01$, $^{***}p<0.001$ significant vs control.

3) Uncropped blots with Citrate-capped AuNPs in MDA-MB-231 cells

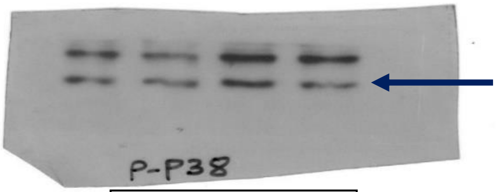

Phospho-p38

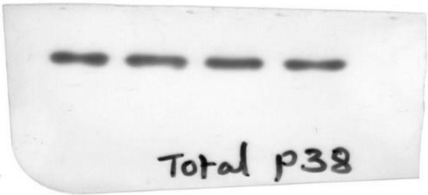

Total p38

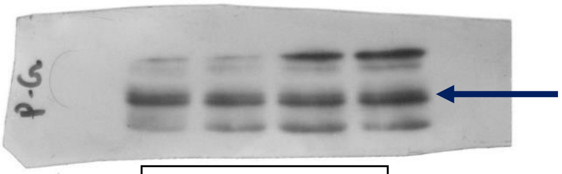

Phospho-GSK-3β

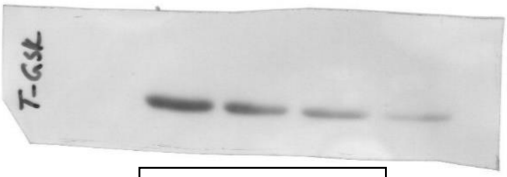

Total GSK-3β

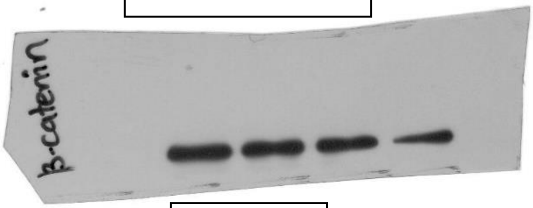

β-catenin

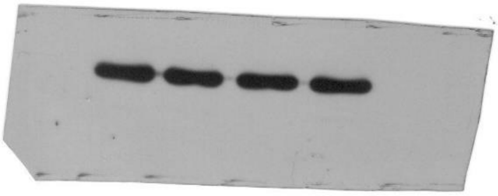

α-tubulin

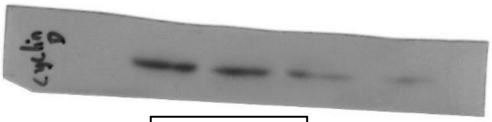

Cyclin D1

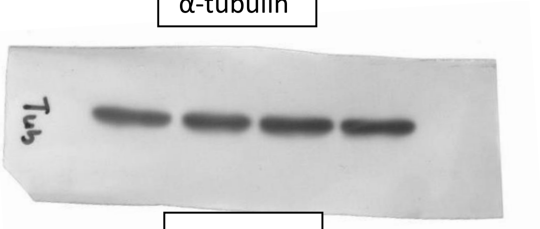

α-tubulin

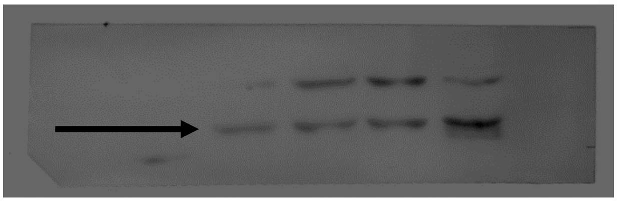

MKP-1

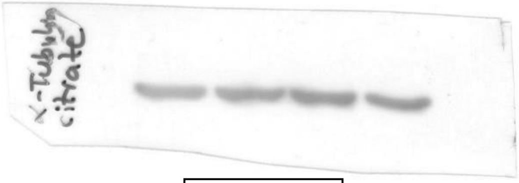

α-tubulin

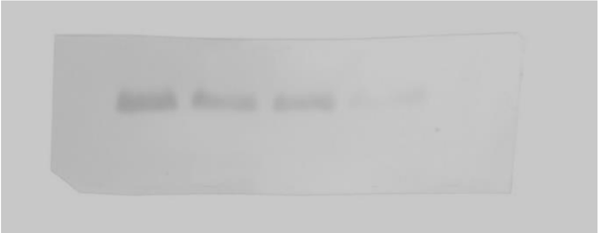

H3-Ser 10 phosphorylation

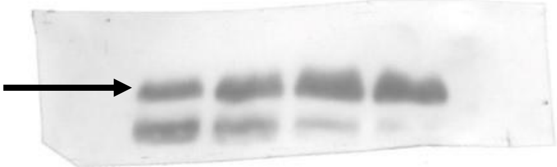

Total H3

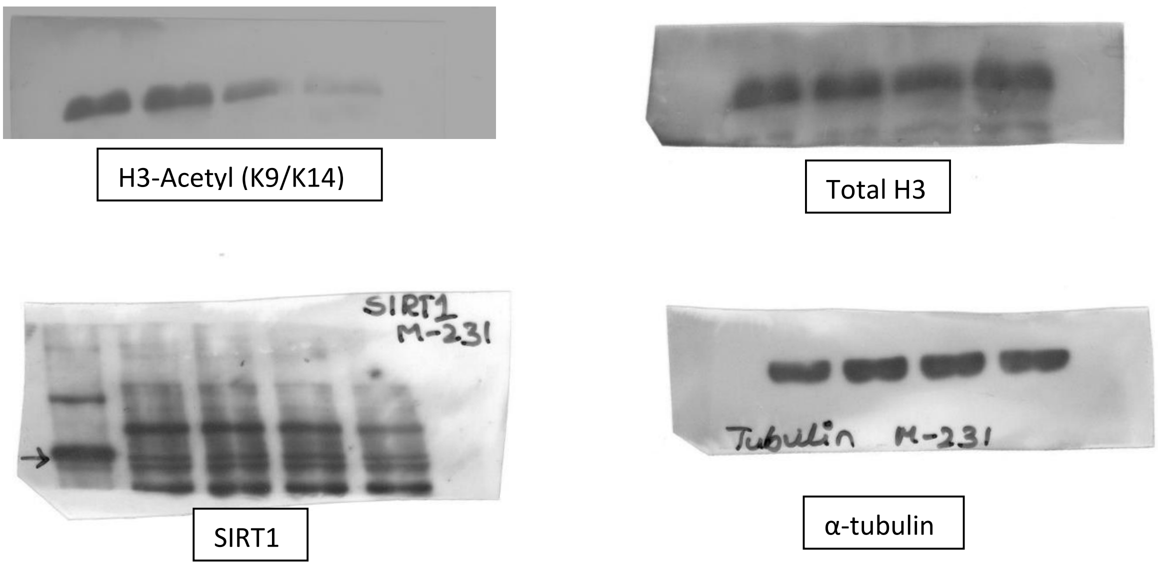

Figure legends: The above pictures represent uncropped images of blots. The first lane in all the blots refer to control group whereas, the second, third and fourth lanes in all blots refer to 100 µg/mL, 250 µg/mL and 500 µg/mL of citrate-capped AuNPs treated groups in MDA-MB-231 cells. Grouped blots of same gel were placed horizontally in the above pictures.

Uncropped blots with cysteamine-capped AuNPs in MDA-MB-231 cells

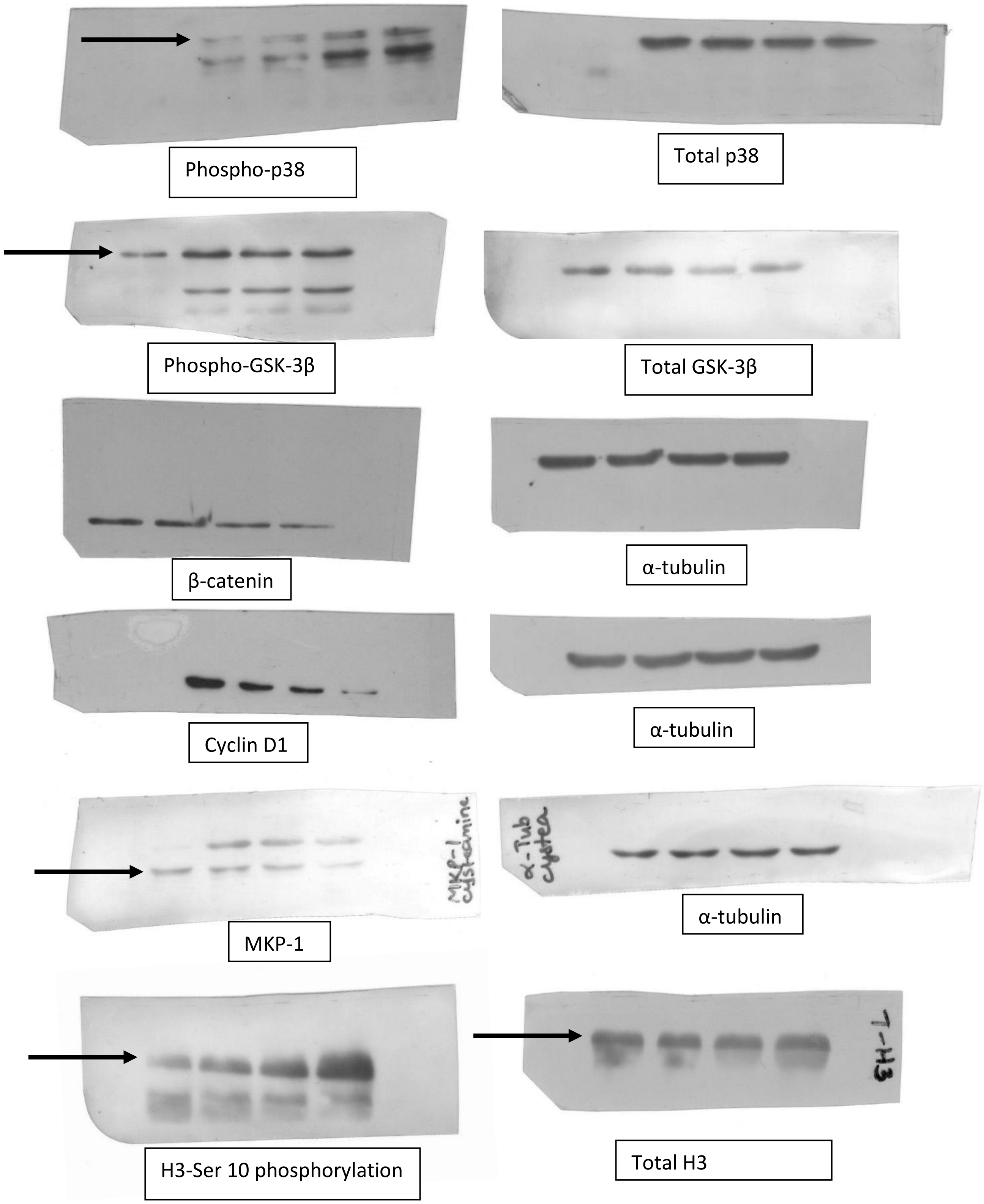

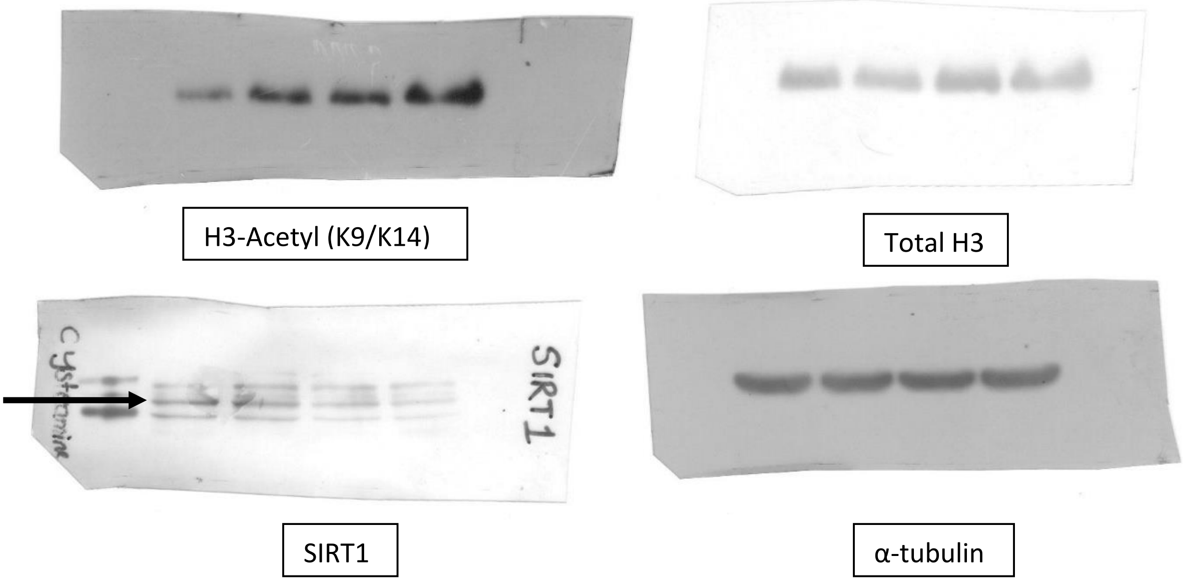

Figure legends: The above pictures represent uncropped images of blots. The first lane in all the blots refer to control group whereas, the second, third and fourth lanes in all blots refer to 100 µg/mL, 250 µg/mL and 500 µg/mL of cysteamine-capped AuNPs treated groups in MDA-MB-231 cells. Grouped blots of same gel were placed horizontally in the above pictures of MDA-MB-231 cells treated with AuNPs.

Uncropped blots with citrate-and cysteamine-capped AuNPs in MDA-MB-468 cells

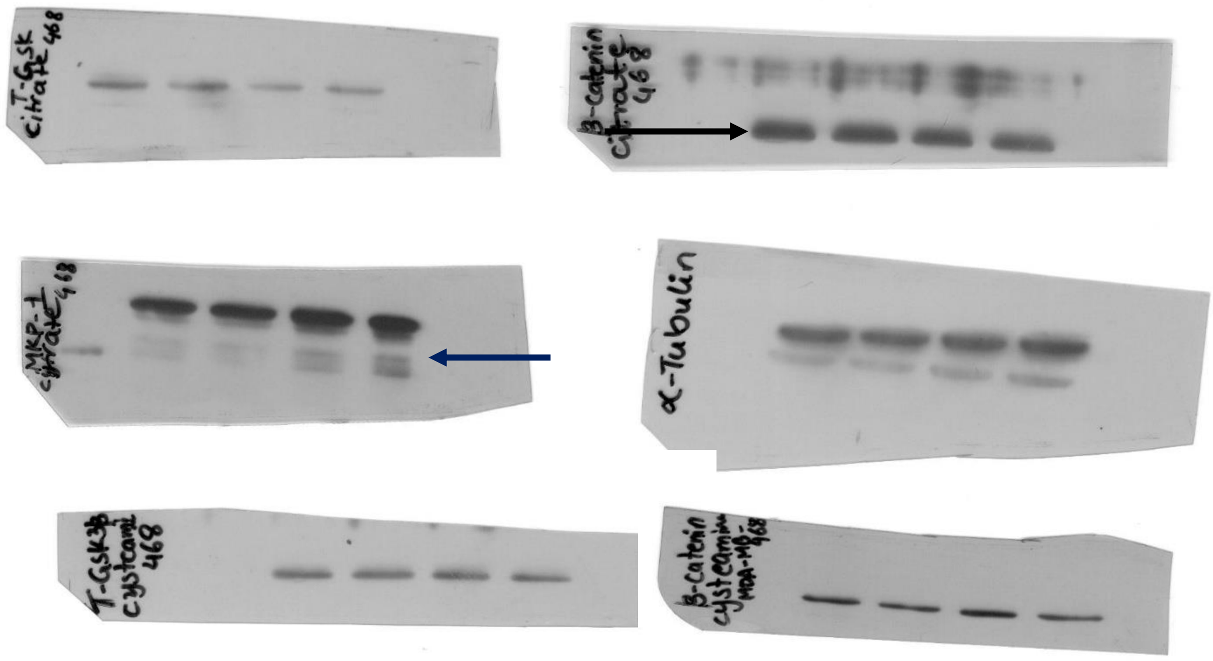

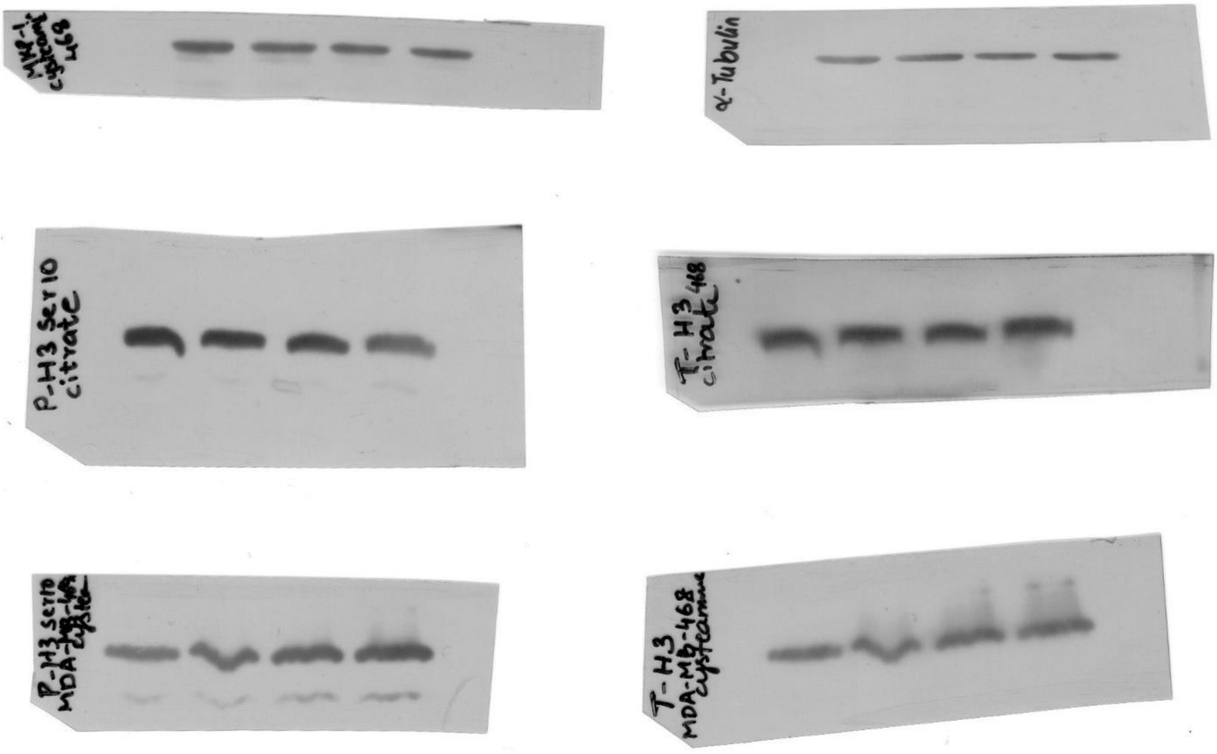

Figure legends: The above pictures represent uncropped images of blots. The first lane in all the blots refer to control group whereas, the second, third and fourth lanes in all blots refer to 100 µg/mL, 250 µg/mL and 500 µg/mL of citrate- and cysteamine-capped AuNPs treated groups in MDA-MB-468 cells. In the case of MDA-MB-468 cells treated with AuNPs, all the blots (GSK-3β, β-catenin, MKP-1 and α-tubulin) shown are from the same gel with the experiments performed separately by both citrate-and cysteamine-capped AuNPs and hence were grouped in supplementary figures 3B and 4B. For histone blots, grouped blots of same gel were placed horizontally in the above pictures of MDA-MB-468 cells treated with AuNPs.

4) References

1. Kimling, J. *et al.* Turkevich method for gold nanoparticle synthesis revisited. *J. Phys. Chem. B.* **110**, 15700-15707 (2006).
2. Vijayakumar, S. & Ganesan, S. In vitro cytotoxicity assay on gold nanoparticles with different stabilizing agents. *J. Nanomater.* **2012**, 14 (2012). http://dx.doi.org/10.1155/2012/734398 (2012).
3. Kim, J.-W., Kim, J. H., Chung, S. J. & Chung, B. H. An operationally simple colorimetric assay of hyaluronidase activity using cationic gold nanoparticles. *Analyst.* **134**, 1291-1293 (2009).
4. Kaur, J. & Tikoo, K. p300/CBP dependent hyperacetylation of histone potentiates anticancer activity of gefitinib nanoparticles. *Biochim. Biophys. Acta.* **1833**, 1028-1040 (2013).
5. Kaur, J. & Tikoo, K. Evaluating cell specific cytotoxicity of differentially charged silver nanoparticles. *Food. Chem. Toxicol.* **51**, 1-14 (2013).

6. Gupta, C., Kaur, J. & Tikoo, K. Regulation of MDA-MB-231 cell proliferation by GSK-3β involves epigenetic modifications under high glucose conditions. *Exp. Cell. Res.* **324**, 75-83 (2014).
7. Amara, V. R., Surapaneni, S. K. & Tikoo, K. Dysregulation of microRNAs and renin-angiotensin system in high salt diet-induced cardiac dysfunction in uninephrectomized rats. *PloS one.* **12**, e0180490 (2017). https://doi.org/10.1371/journal.pone.0180490 (2017).
